# Breast cancer involvement of the nipple-areola complex and implications for nipple-sparing mastectomies: a retrospective observational study in 137 patients

**DOI:** 10.1186/s13037-019-0191-7

**Published:** 2019-03-16

**Authors:** Mohammed Faisal, Hamada Fathy, Ahmed M. M. Gomaa, Haidi Abd-Elzaher, Mohamed A. H. Ahmed, Mohamed Gamal Sayed

**Affiliations:** 1 Surgical Oncology Unit, Department of Surgery, Faculty of Medicine, Suez Canal University Hospital, Circular Road, Ismailia, 411522 Egypt; 2 Department of Pathology, Faculty of Medicine, Suez Canal University, Ismailia, Egypt

**Keywords:** Nipple-sparing mastectomy, NAC, Breast cancer

## Abstract

**Introduction:**

Nipple-sparing mastectomy (NSM) has gained much attention by enhancing the aesthetic outcome in breast carcinoma patients. The aim of this study was to assess the prevalence of malignant affection of the nipple-areola complex (NAC) in breast carcinoma patients and its correlation with prognostic factors for breast cancer.

**Patients and methods:**

This study included 137 female patients diagnosed with breast carcinoma at different disease stages who were admitted to our surgical oncology unit at Suez Canal University Hospital from June 15, 2014 to January 25, 2017. We excluded patients with evidence of nipple involvement as ulceration or patients with previous breast surgery with periareolar incisions. This study was designed to test the hypothesis that the NAC can be spared in certain selected patients. All studied participants provided a full history and underwent general and local clinical examinations, pre-operative laboratory tests, and radiological and pathological evaluations.

**Results:**

The mean age of the study population was 47.39 ± 8.01 years. Among the patients, the NAC was affected in 12 (11.40%) patients. Patients with NAC involvement showed a significantly larger tumor size of more than 4 cm and a shorter tumor-nipple distance of less than 2 cm (*p* = 0.000). Lymph node metastasis was associated with NAC involvement (*p* = 0.001), with increased risk when more than 10 lymph nodes were involved (*p* = 0.007). Lymphovascular invasion was a significant predictor of NAC involvement (*p* = 0.014). Multifocal as well as multicentric tumors were significantly associated with NAC involvement (*p* = 0.016 and 0.003, respectively). NAC involvement was more likely in Estrogen receptor (ER) and Progesterone receptor (PR) patients than in ER+ and PR+ patients (*p* = 0.000), while Human epidermal receptor (HER+) patients were more likely to have NAC involvement than HER patients (p = 0.000). Additionally, stage ΙΙΙ cancer was significantly associated with NAC involvement (*p* = 0.041), and histological grade III disease carried a greater risk than grade I disease of NAC involvement (*p* = 0.008).

**Conclusion:**

The incidence of NAC affection among breast carcinoma patients who underwent mastectomy and axillary clearance was associated with important parameters, such as tumor size, areola edge-tumor distance, lymph node affection, hormonal receptor status and lymphovascular invasion. Accordingly, NAC-preserving surgeries could be tailored to patients with favourable tumor characteristics.

## Introduction

There has been significant progress in the operative management of breast carcinoma within the last six decades, as demonstrated by the adoption of conservative breast cancer surgery with great enhancements in early and delayed breast reconstruction surgery into skin-sparing mastectomy, which maintains an original skin cover and has already gained broad approval [1, 2].

Despite this progress, conventional skin-sparing mastectomy forfeits both the nipple and the areola, which serve as a focus of the breast [2]. The nipple-areola complex (NAC) provides unique character because many women believe that their own breast renovation is not complete until the nipple is reassembled [3]. Nonetheless, nipple renovation rarely provides patient satisfaction because of insufficient projection, colour, form, sizing, texture, consistency and posture [4, 5].

Nonetheless, nipple renovation rarely provides patient satisfaction because of insufficient projection, colour, form, sizing, texture and consistency and posture [6].Also Nipple reconstruction is at risk of tissue necrosis as the tissue becomes critically ischemic when raised off the skin blood supply. Skin perfusion is related to microcirculation [7] As such, nipple-sparing mastectomy (NSM) has gained consideration for the purpose of achieving 3 main targets: first, oncological safety; second, nipple-areola viability; and third, improved outcome [8]. By far, the most crucial of these aims is to achieve oncological safety because the nipple is linked to the mammary gland through lactiferous ducts and is likely at risk for malignant locoregional recurrence.

Recent multivariate models that can help the selection of patients eligible for NSM have revealed the following predictors of occult nipple involvement: tumour size, tumour location and tumour-nipple distance [9].

## Patients and methods

### Study design

A retrospective observational study that took place at the Surgical Oncology Unit of the Department of Surgery, Suez Canal University Hospital, from June 15, 2014 to January 25, 2017 assessing the prevalence of malignant affection of the nipple-areola complex (NAC) in breast carcinoma patients and its correlation with prognostic factors for breast cancer. This research project has been approved by the research ethics committee of the Faculty of Medicine, Suez Canal University (reference number #1890.

### Study population

The study included One hundred thirty-seven (137) women with a diagnosis of operable breast cancer who underwent modified radical mastectomy. We excluded patients with clinical involvement of the NAC, such as Paget’s disease, fixed nipple, and nipple erosion or induration, scheduled for other simultaneous procedure, had recurrent breast cancer patient, with previous radiation over chest wall, who cannot understand or cannot accept the study (refused to sign written informed consent) or patient unfit for surgery. Written consent was obtained from all patients.

### Study hypothesis

We hypothesized that nipple- sparing mastectomy can be performed in certain selected patients with oncological safety and better cosmetic outcome.

### Study outcome

- The primary outcome was to identify the subtle group of patients whom will have a privilege of NAC-sparing mastectomy.
- The secondary outcome of this study was to correlate between NAC involvement and certain tumor factors including tumor size, tumor–NAC distance, histological type, nuclear grade of the tumor and axillary lymph node status.

### Preoperative workup and surgical procedure

All patients provided a full history and underwent general and local clinical examinations, pre-operative laboratory tests, and radiological and pathological evaluations, pre-operative needle biopsy, either fine-needle aspiration biopsy (FNAB) or Tru-Cut core needle biopsy guided by ultrasonography.

### Study intervention and surgical procedure

The nipple and areola were segregated from the breast tissue and then sectioned at 5-mm intervals from the core of the nipple to the edge. Each section was further sectioned at 2.5 mm and embedded for assessment of its entirety. Specimens were examined histopathologically, as follow: Tumor size, type, and grade, Lymph node involvement, number of involved lymph nodes, tumor stage, and NAC involvement, as indicated by malignant cells. Malignant NAC involvement was examined in detail, including in terms of the distance from the periphery of the main tumour. All patients enrolled in this study were managed by modified radical mastectomy. The breast tissue was removed, and formal axillary lymph node dissection was performed through an elliptical incision encompassing the NAC. All breast tissue and axillary lymph nodes were removed en bloc, followed by histopathological examination.

### Statistical analysis

SPSS version 20 (IBM) was used for the data analysis; 95% confidence intervals (CIs) were calculated, and *p* values < 0.05 were considered statistically significant. Data. Mann Whitney was used for non-normally distributed data of quantitative variables. Fischer exact test was used to compare between data of qualitative nominal variables. Wilcoxon sign test was used compare between data of qualitative categorical variables. Relative Risks (RRs) with 95% confidence intervals (CIs) had been estimated from the novel publications. To measure the pooled RRs with 95% CIs, fixed-effect model was used when there was minimal heterogeneity in the variables among studies and random-effect-model when significant heterogeneity. Between-study, chi-square test was used for presenting heterogeneity of RR. Chi Square test was considered significant if p<0.05. ROC curve analysis was performed to identify a cut-off value for the tumour-nipple distance associated with NAC involvement.

## Results

**Fig. 1:**
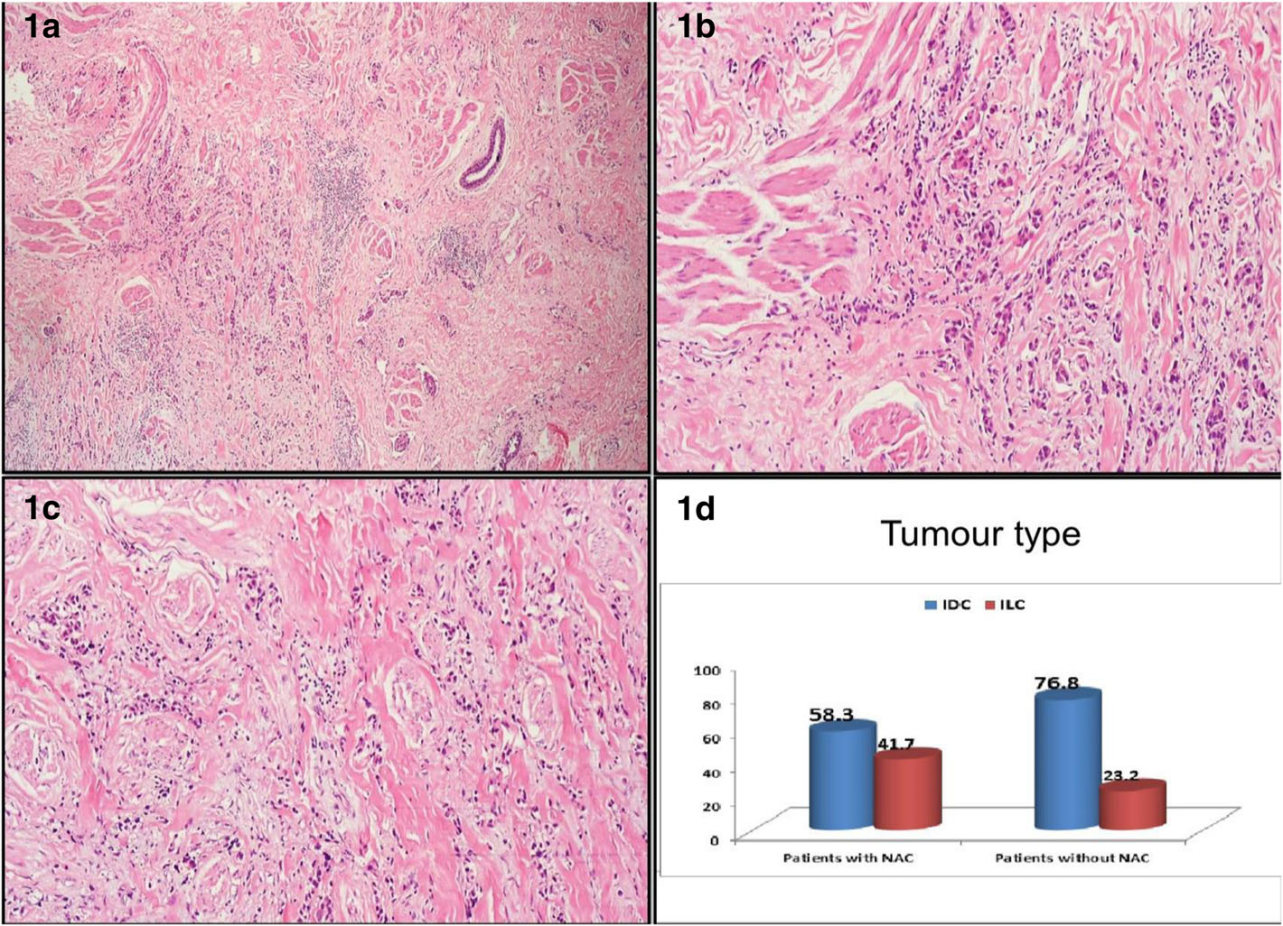
Invasive ductal carcinoma of no special type surrounding the smooth muscle bundles of the nipple-areola complex (100X) (**a**). Higher magnification of (**a**) (200X) (**b**). Invasive lobular carcinoma surrounding the smooth muscle bundles of the nipple-areola complex (100X) (**c**). Distribution of the tumour types included in the study in relation to nipple-areola complex involvement (**d**)

In the present study, 137 female patients with breast cancer were included. They were classified after mastectomy into 2 subgroups according to NAC involvement. Demographic and clinicopathological data for all patients are shown in Table 1. The upper outer compartment was the most common tumor site in all patients (37.2%). Regarding histopathology, the most common type of all recruited patients was invasive ductal carcinoma (IDC, 75.2%), followed by infiltrating lobular carcinoma (ILC, 24.2%), with 46% of the patients having histological grade Ι disease. Lymph node involvement was present in 28.5% of all patients. Stage ΙΙ disease was present in the majority of cases (58.4%). Lymphovascular invasion was revealed in 15.3% of the patients Fig 1.

**Table 1:** Demographic and clinicopathologic data of all patients

		All patients	Patients with NAC *N* = 12	Patients without NAC *N* = 125	*p*-value
Age		47.39 ± 8.01	50.9 ± 6.8	47.1 ± 8.1	0.136 ^a^
		32–58			
Age subgroups	30–39	35 (25.5)	2 (16.7)	33 (26.4)	0.686 ^b^
	40–49	37 (27)	3 (25)	34 (27.2)	
	≥ 50	65 (47.4)	7 (58.3)	58 (46.4)	
Tumor size		2.9 ± 1.16	4.18 ± 1.16	2.35 ± 1.1	<0.001 ^a^
Range		1–6			
< 4 cm		101 (73.7)	4 (33.3)	97 (77.6)	0.003 ^b^
≥ 4 cm		36 (26.3)	8 (66.7)	28 (22.4)	
Tumor nipple distance		3.38 ± 0.92	1.95 ± 0.72	3.52 ± 0.84	<0.001^a^
Range		1.4–6			
< 2 cm		6 (4.4)	4 (33.3)	2 (1.6)	<0.001 ^ɑ^
2 – < 2.5 cm		11 (8)	2 (16.7)	9 (7.2)	
2.5 - < 3 cm		4 (2.9)	3 (25)	1 (0.8)	
3 - < 4 cm		79 (57.7)	1 (8.3)	78 (62.4)	
≥ 4 cm		37 (27)	2 (16.7)	35 (28)	
Tumor site	Upper inner	22 (16.1)	1 (8.3)	21 (16.8)	0.470 ^b^
	Upper outer	26 (19.1)	2 (16.7)	24 (19.2)	
	Lower inner	31 (22.6)	1 (8.3)	30 (24)	
	Lower outer	7 (5.1)	1 (8.3)	6 (4.8)	
	Central	51 (37.2)	7 (58.3)	44 (35.2)	
Histologic type	DCIS	32 (23.4)	5 (41.7)	27 (21.6)	0.033 ^b^
	IDC	37 (27)	5 (41.7)	32 (25.6)	
	LCIS	43 (31.4)	1 (8.3)	42 (33.6)	
	ILC	25 (18.2)	1 (8.3)	24 (19.2)	
histologic grade	Grade I	63 (46)	2 (16.7)	61 (48.8)	0.003 ^b^
	Grade II	39 (28.5)	2 (16.7)	37 (29.6)	
	Grade III	35 (25.5)	8 (66.7)	27 (21.6)	
Lymph node involvement	Positive	39 (28.5)	9 (75)	30 (24)	0.001^b^
Lymph node numbers	< 4	13 (9.5)	0 (8.3)	13 (43.3)	<0.001 ^ɑ^
	4 - < 10	14 (10.2)	1 (11.1)	13 (43.3)	
	≥ 10	12 (8.8)	8 (88.9)	4 (13.3)	
Staging	II	80 (58.4)	3 (27.3)	77 (61.6)	0.032 ^b^
	III	57 (41.6)	8 (72.7)	49 (38.9)	
Multifocal tumor		13 (9.5)	4 (33.3)	9 (7.2)	0.016 ^b^
Multicenteric		14 (10.2)	5 (41.7)	9 (7.2)	0.003 ^b^
Lymphovascular invasion		21 (15.3)	5 (41.7)	16 (12.8)	0.020 ^b^
Estrogen receptor status		13 (9.5)	6 (50)	7 (5.6)	<0.001^b^
Progesterone receptor status		12 (8.8)	7 (58.3)	5 (4)	<0.001^b^
HER status		17 (12.4)	7 (58.3)	10 (8)	<0.001^b^

^a^:Mann Whitney test ^b^: Fischer exact test ^ɑ^: Wilcoxon sign test

While comparing the subgroups regarding age revealed no significant differences, patients with NAC involvement showed significantly larger tumors and shorter tumor-nipple distances than patients without NAC involvement. There was no difference in the tumor site between the subgroups. There were no significant differences between the subgroups regarding the histological type; however, regarding the histological grade, grade ΙΙΙ disease was significantly more frequent among the patients with NAC involvement.

Lymph node involvement was significantly more common in the NAC group, as more than 10 lymph nodes were excised from approximately 89% of patients in this subgroup compared to 13.3% of patients in the other subgroup. Stage ΙΙΙ disease was found in 72.7% of patients with NAC involvement compared to 38.9% of patients without NAC involvement (*p* = 0.032). Multicentric and multifocal tumors were detected significantly more frequently among patients with NAC involvement (*p* = 0.016 and 0.003, respectively), as was lymphovascular invasion, which was found in 41.7% of patients with NAC involvement compared to 12.8% of patients without NAC involvement. A significant number of patients with NAC involvement were ER- and PR-, but a higher percentage of patients in this group than in the other group were HER+.

Age was not a significant predictor of NAC involvement. The risk of NAC increased with tumor sizes ≥4 cm compared to tumor sizes < 4 cm (RR = 6.92, *p* = 0.003). Tumor-nipple distances < 2 cm versus > 2 cm and <  2.5 cm versus > 2.5 cm carried a greater risk of NAC involvement Relative Risk (RR) =0.029 and 0.019; *p* = 0.002 and 0.004, respectively) Table 2.

**Table 2:** Predictors of NAC involvement

		RR	CI (95%)		Wald	p-value
			Lower	upper		
Age subgroups	< 40 versus > 40 years	1.99	0.391	10.14	0.687	0.407
	> 40 versus > 50 years	1.36	0.332	5.64	0.188	0.665
Tumor size < 4 versus ≥ 4 cm		6.92	1.94	24.17	8.89	0.003
Tumor nipple distance < 3 cm	< 2 versus > 2 cm	0.029	0.003	0.262	9.88	0.002
	< 2.5 versus > 2.5 cm	0.019	0.001	0.276	8.42	0.004
	< 3 versus > 3 cm	0.257	0.032	2.08	1.61	0.203
	< 4 versus > 4 cm	4.45	0.391	50.79	1.44	0.229
Lymph node metastasis	Metastasis versus no metastasis	9.5	2.41	37.37	10.37	<0.001
	≥ 10versus < 10	26.00	2.45	275.82	7.31	0.007
Multifocal tumor	Multifocal versus not multifocal	6.44	1.62	25.58	7.01	0.008
Multicenteric	Multicentric versus not multicentric	9.20	2.42	34.91	10.65	<0.001
Lymphovascular invasion	Invasion versus noninvasion	4.86	1.37	17.18	6.04	0.014
Estrogen receptor status	ER - versus ER+	16.85	4.30	65.97	16.46	<0.001
Progesterone receptor status	PR - versus PR+	33.60	7.84	143.97	22.40	<0.001
HER status	HER + versus HER -	16.0	4.31	60.09	17.10	<0.001
Staging III	Stage III versus II	4.19	1.1	16.56	4.17	0.041
Histologic tumor type	DCIS versus ILC	0.225	0.025	2.06	1.74	0.187
	DCIS versus IDC	0.267	0.029	2.43	1.37	0.241
	IDC versus ILC	1.75	0.105	29.26	0.152	0.697
Histologic grade	Grade III versus grade I	9.03	1.79	45.40	7.14	0.008
	Grade III versus grade II	5.48	1.07	27.89	4.20	0.040

Lymph node metastasis was associated with NAC involvement (RR = 9.5; *p* = 0.001), with increased risk when more than 10 lymph nodes were involved (RR = 26; *p* = 0.007). Lymphovascular invasion was a significant predictor of NAC involvement (RR = 4.86; *p* = 0.014). Multifocal (RR = 6.44; *p* = 0.008) and multicentric (RR = 9.5; p = 0.001) tumors were significantly associated with NAC involvement Table 2.

ER- patients had a higher risk for NAC involvement than ER+ patients (RR = 16.85; *p* < 0.001). Additionally, PR- patients had a higher risk for NAC involvement than PR+ patients (RR = 33.6; p < 0.001) Table 2.

HER+ patients were more likely to have NAC involvement than HER- patients (RR = 16.0; p < 0.001). Stage ΙΙΙ cancer was significantly associated with NAC involvement compared to stage ΙΙ cancer (RR = 4.19; *p* = 0.041) Table 2.

Both histopathological types identified were considered non-predictors for NAC; however, histological grade III disease carried a greater risk than grade I disease of developing NAC involvement (RR = 9.03; *p* = 0.008). Additionally, histological grade III disease carried a greater risk than grade II disease of developing NAC involvement (RR = 5.48; *p* = 0.040) Table 2.

**Fig. 2:**
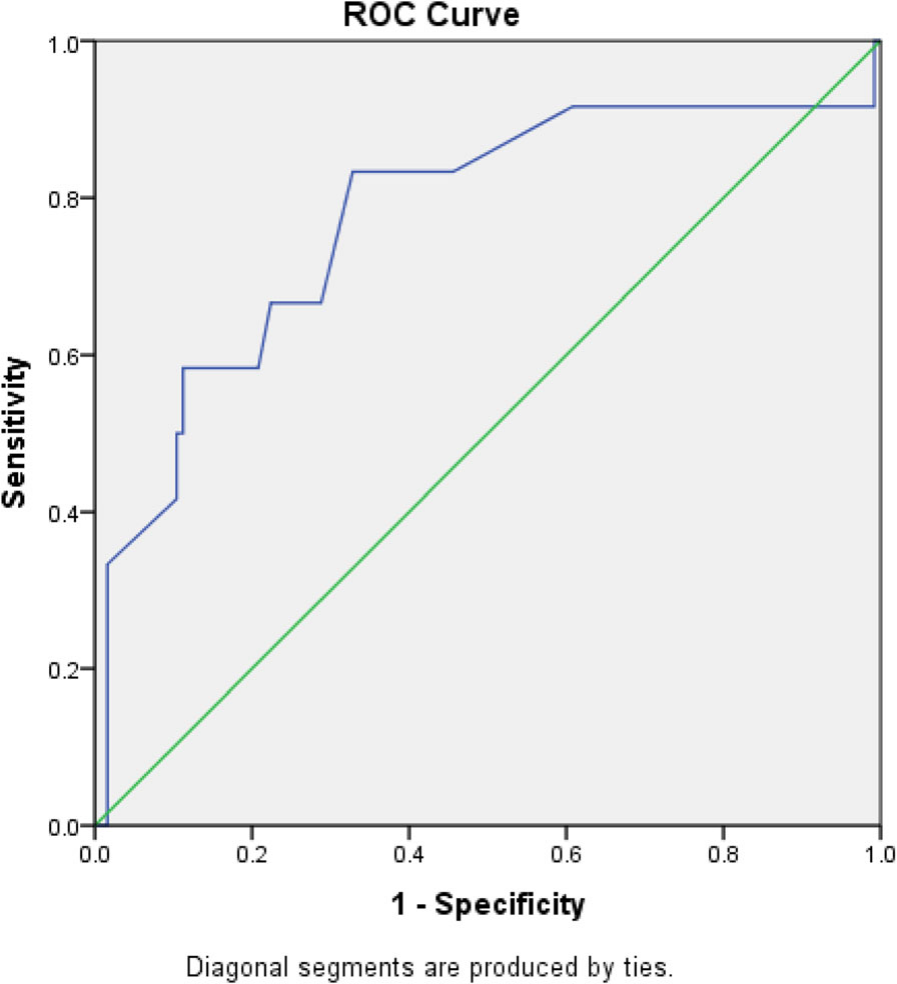
ROC curve, cutoff value of tumor nipple distance for detecting NAC

ROC curve analysis was performed to identify a cut-off value for the tumor-nipple distance associated with NAC involvement; a tumor-nipple distance of 2.4 cm had a sensitivity and specificity of 83 and 68%, respectively (AUC = 0.775; *p* = 0.002) Fig. 2.

## Discussion

NAC resection was previously included in the surgical management of breast carcinoma. This was done regarding the evidence of accumulated lymphatic drainage of the breast into the subareolar Sappey plexus before eventually draining into the axillary lymph nodes [10]. Turner-Warwick had discarded the previous theory and demonstrated that lymphatic vessels accompany blood vessels in the breast tissue leaving the nipple area [11]. To clarify this discrepancy, we postulated that Sappey’s original studies included lactating but not resting breasts [12].

In this analysis, we studied surgical specimens from 137 females who had undergone mastectomy. Of all the examined specimens, 12 (8.7%) showed tumour involvement in the NAC. Six decades ago, some authors reported rates of tumor involvement in the NAC higher than that found in this study [13–15]. A relatively high rate of NAC involvement was also documented in a study by Pirozzi et al. [16]; however, only 50 women were enrolled in their study. Another study reported only 22 cases (16%) of NAC involvement [17]. A small Libyan study documented NAC involvement in only 3 (12%) out of 25 cases [18], which is similar to the figure reported by Crowseet et al. [19], while an Egyptian study demonstrated an incidence rate of 6.3% [20]. Incidence rates of nipple involvement (NI) ranging from 0 to 58% have been reported recently [21]; however, that study did not include the areola in the analysis. The anatomical difference between the areola and nipple is that the areola does not contain parenchymal ducts; on the other hand, the nipple has a lining of ductal cells; any of those cells could potentially produce breast cancer.

Many women prefer to preserve the nipple after mastectomy for a better cosmetic outcome. For this reason, multiple studies, including ours, have investigated the differences between breast cancer patients with and without NAC involvement and demonstrated the predictors of NAC involvement to enable the selection of patients who may be candidates for NAC preservation while maintaining oncological safety; however, other studies have shown discrepancies regarding the predictive factors.

In our study, we detected significantly larger tumours with shorter tumour-nipple distances in patients with NAC involvement than in patients without NAC involvement. Patients with a tumour-nipple distance ≤2.5 cm were more likely to have NAC involvement than patients with longer distances; additionally, patients with a tumour size ≥4 cm were at risk for NAC involvement (*p* = 0.003). A large meta-analysis by Zhang et al. reported that tumour-to-nipple distances ≥2.5 cm were associated with a reduced risk of NI [22]. Other reports have confirmed this distance regarding NI [17, 23]. Although another study comparing 2 groups regarding NAC involvement revealed no difference according to the tumor-nipple distance (*p* = 0.735), in the same study, when a dichotomous division of the distance was analysed, a distance ≤3 cm was detected in 100% of cases with NAC involvement compared to 60.5% of cases without NAC involvement (*p* = 0.007) [16]. This finding also agrees with the data reported by Bishop et al. [24]. A tumour size of more than 5 cm carried an increased risk for NI in a study by Wang et al. [25]. Pirozzi and his colleagues reported findings consistent with our results with respect to tumour size, as they found no differences between the two studied groups regarding NI when they categorized tumour size as < 3 or ≥ 3 cm (*p* = 0.693) [16].

With respect to lymph node metastasis in our analysis, we found a significantly higher rate of lymph node involvement among patients with NAC involvement than those without NAC involvement (9(75%) versus 30(24%), respectively, *p* = 0.001). Additionally, lymphovascular invasion was more prevalent in patients with than in those without NAC involvement (5(41.7%) vs. 16 (12.8%), respectively). Mallon et al. described that nodal involvement was a valuable predictor of NAC involvement [26]; however, another report revealed contrasting results [27], in which a higher nodal stage was not associated with NI. The results reported by Wang et al. agree with our findings, as they demonstrated that nodal involvement was associated with NAC involvement (NAC involvement in 14% of tumours positive for nodal involvement vs. 8% in tumour negative for nodal involvement, *p* = 0.0331) [25].

Despite lymphovascular invasion being an important predictor of increased tumour aggressiveness, the data published by Pirozzi et al. revealed that lymphovascular invasion was detected in 15 of the 50 cases studied; of those patients, 14 did not show NI, and only 1 showed NI. Comparing the two groups showed no significant difference [16]. The previous results were in contrast with those revealed by Vyas et al. [17] and Vlajcic et al. [28], probably owing to the small sample size included in the current study.

Regarding the histological type among our cases, we detected that among all patients, as well as patients with NAC involvement, there was a predominance of IDC over ILC; however, there was no significant difference (*p* = 0.171). Additionally, none of the detected pathological types were significant predictors of NAC involvement (*p* = 0.167). However, the histological grade was considered a significant predictor of NAC involvement among our cases, as 8 (66.7%) of the patients with NAC involvement had grade 3 tumors.

Multiple studies have suggested that NI is associated mainly with ductal carcinoma in situ (DCIS) [23, 29–31]. Li et al. [32] demonstrated that IDC or IDC with associated DCIS and breast carcinoma with invasive micropapillary carcinoma (IMPC) components were the most common tumour types associated with NI. Contrasting results regarding the histological grade were reported by Pirozzi et al., as they found no significant difference among groups regarding the histological grade [16]. Other studies have suggested that lobular carcinoma in situ (LCIS) is strongly associated with NI [33], although others have reported a negative association between LCIS and NI [34], as LCIS is not considered a true tumor lesion and does not require an additional safety margin when present at the surgical margin [35].

In our study, there was no significant difference in the tumor site between the groups, meaning that no specific region was associated with NAC involvement. Multiple studies have shown that the tumor location is an important predictor of NAC involvement [12, 36, 37]. This discrepancy could be attributed to the relatively small sample size, which is also reflected in the relatively low positive rate of NAC involvement.

In our study, 6(50%) of patients with NAC involvement were ER-, while 7(58.3%) of patients with NAC involvement were PR- and HER+. A systematic review by Zhang et al. reported a higher rate of NI among ER- patients (1.189, 95% CI = 1.008–1.404) and PR- patients (1.515, 95% CI = 1.248–1.84). On the other hand, the pooled analysis of the same study with respect to HER status revealed a stronger association between having NI and being HER+ (RR = 1.760, 95% CI = 1.463–2.116) [22]. Another study reported that HER overexpression was associated with NAC involvement (*p* = 0.0137), which supports our findings [25]. Brachtel et al. have also reported that HER2 overexpression is associated with NAC involvement [31]. HER positivity in patients with NAC involvement may be a potential indicator of the presence of Paget’s disease. Pirozzi and his colleagues published different results regarding hormonal factors, as they found no significant differences between the groups described in this study regarding PR and ER status (*p* = 0.794 and 0.825, respectively) [16].

## Conclusion

The incidence of NAC involvement among patients with breast carcinoma who underwent mastectomy and axillary clearance was associated with certain important factors, such as the tumour size, the distance from the tumour site to the edge of the areola, lymph node metastasis, lymphovascular invasion, HER overexpression, and ER and PR negativity. Accordingly, NAC-preserving surgeries and NSM are oncologically safe procedures in well selected patients with tumors at stages 0–II, peripheral tumors far from the nipple, and with favourable pathological features.

## Abbreviations

CI: Confidence Incidence
DCIS: Ductal carcinoma in situ
ER: Estrogen receptor
HER: Human epidermal receptor
LBCI: Lobular carcinoma in situ
NAC: Nipple Areola Complex
NI: Nipple Involvement
NSM: Nipple sparing mastectomy
PR: Progesterone receptor
RR: Relative Risk
